# Pattern and severity of multimorbidity among patients attending primary care settings in Odisha, India

**DOI:** 10.1371/journal.pone.0183966

**Published:** 2017-09-14

**Authors:** Sanghamitra Pati, Subhashisa Swain, Job Metsemakers, J. André Knottnerus, Marjan van den Akker

**Affiliations:** 1 Public Health Foundation of India, Indian Institute of Public Health, Bhubaneswar, Odisha, India; 2 Dept Family Medicine, School Caphri, Maastricht University, Maastricht, The Netherlands; 3 Dept General Practice, KU Leuven, Leuven, Belgium; The Chinese University of Hong Kong, HONG KONG

## Abstract

Multimorbidity is increasingly the primary concern of healthcare systems globally with substantial implications for patient outcomes and resource cost. A critical knowledge gap exists as to the magnitude of multimorbidity in primary care practice in low and middle income countries with available information limited to prevalence. In India, primary care forms the bulk of the health care delivery being provided through both public (community health center) and private general practice setting. We undertook a study to identify multimorbidity patterns and relate these patterns to severity among primary care attendees in Odisha state of India. A total of 1649 patients attending 40 primary care facilities were interviewed using a structured multimorbidity assessment questionnaire. Multimorbidity patterns (dyad and triad) were identified for 21 chronic conditions, functional limitation was assessed as a proxy measure of severity and the mean severity score for each pattern, was determined after adjusting for age. The leading dyads in younger age group i.e. 18–29 years were acid peptic disease with arthritis/ chronic back ache/tuberculosis /chronic lung disease, while older age groups had more frequent combinations of hypertension + arthritis/ chronic lung disease/vision difficulty, and arthritis + chronic back ache. The triad of acid peptic disease + arthritis + chronic backache was common in men in all age groups. Tuberculosis and lung diseases were associated with significantly higher age-adjusted mean severity score (poorer functional ability). Among men, arthritis, chronic backache, chronic lung disease and vision impairment were observed to have highest severity) whereas women reported higher severity for combinations of hypertension, chronic back ache and arthritis. Given the paucity of studies on multimorbidity patterns in low and middle income countries, future studies should seek to assess the reproducibility of our findings in other populations and settings. Another task is the potential implications of different multimorbidity clusters for designing care protocols, as currently the protocols are disease specific, hardly taking comorbidity into account.

## Introduction

With increased life expectancy and better living conditions, the co-occurrence of multiple chronic conditions, commonly referred to as ‘multimorbidity’ is being increasingly observed among individuals globally [1]. Compared to comorbidity, multimorbidity, the concurrent presence of two or more chronic conditions is independent of an underlying or index disease [2]. The consequences of multimorbidity are manifold—individuals with multiple morbidities experience an inferior quality of life, a poorer perception of their physical and mental health, experience higher in-hospital mortality, longer length of hospital stay, incur higher healthcare expenditure, and a lower functional capacity[3–5]. Evidence suggests that it is not the chronic conditions by themselves that increase the resource cost. It is the number of types of conditions, i.e multimorbidity which is responsible [6,7]The current knowledge of multimorbidity is largely based on studies from western countries demonstrating the prevalence to be high ranging from 30% in all adults to 60% among 65–74 year olds [8]. Moreover, initially thought to be a geriatric phenomenon, several recent studies have demonstrated that younger adults also exhibit a considerable prevalence of multimorbidity[9]. Ironically, existing care models and the clinical guidelines for chronically ill patients are mostly focused on management of single conditions separately though diseases are rarely presented in isolation[10].

Despite some conceptual differences, the most commonly used approach to express the magnitude of multimorbidity has been simple counting of chronic conditions which provides a measure of prevalence or use of indices like Charlston Comorbidity Index, which provides more or less a weighted score [11]. However, multimorbidity is a complex phenomenon with a labyrinth full of possible disease combinations; therefore, the use of a quantitative definition has been used extensively to capture the patterns of diseases. The co-occurrence of diseases has implications from a disease management point of view; the impact of multiple diseases may compound and interact, and the summative features can be much more complicated than a simple aggregation of individual illnesses. Since, the negative outcomes associated with multimorbidity are largely attributable to the clustering and typology of conditions, the coexistence rather than the number is a matter of concern[6]. Thus, information on only prevalence by simple count may not be sufficient enough to guide primary health services design; it is additionally important to examine the patterns for appropriate clinical management and adequate understanding of the disease effect by researchers and policymakers.

Besides count and combinations of constituent conditions, the burden of multimorbidity among those affected depends on the severity of chronic conditions, temporal evolution and social factors as well [12,13]. Groups of conditions may have a synergistic effect on functional ability, and specific diseases may be associated with difficulties in different functional tasks, a complexity not captured through one-dimensional (simple count) or two-dimensional (count and combinations) indices. Identifying particular combinations of diseases that have highest effect on functional limitation can provide health and social care professionals with insights on synergies and effects associated with coexisting diseases and design services accordingly[10]. Multidimensional indices that have incorporated severity (measured by functional limitation) to estimate the magnitude multimorbidity have shown to be correlated well with health outcomes including quality of life and mortality [6,14]. The recent global move on primary care-led management of NCDs has emphasized the importance of patterns analysis in the multimorbidity research agenda. Yet, there are a far smaller number of studies assessing the severity or functional limitation dimension of multimorbidity.

Surprisingly, especially in low and middle income countries, multimorbidity is an underexplored domain till date despite the rising prevalence of NCDs in these countries[8]. Little research has investigated this entity in India, the second largest demography in the world. Our recent systematic review has revealed the rudimentary work on multimorbidity in South Asia with conspicuous absence of any reports from Indian primary care[15]. Available estimates mostly describe the prevalence confined to either a limited set of chronic conditions or specific age groups. More so, no study had examined the patterns or severity within the ambit of multiple morbidities thus potentially leading to inadequate information for care management. It is not yet known how multimorbidity is distributed across age and sex group and what are the commonly occurring combinations of chronic diseases in individuals and their outcomes. This information is critical as traditionally, the health care systems in India have been configured around the ideology of the infectious diseases and singular-morbidity oriented care[16,17]. Thus, multimorbidity could be easily ignored by the clinician at the same time being challenging to address. Primary care physicians are the first point of contact and the main providers of healthcare in the country. Given their continuity of care and coordinating role, these settings are well suited for managing multiple chronic diseases. We therefore undertook a study to examine the pattern and severity of multimorbidity in primary care patient population in an Indian state having health indicators similar to the national average. The objectives were—to describe the multimorbidity patterns, identify the commonly occurring combinations (dyads and triads), analyze the age and gender-specific differences in the patterns and assess functional limitation or severity across different patterns. It is expected that our findings would provide primary care clinicians and policy makers with better clinical and epidemiological understanding of multimorbidity for aligning care management.

Our research questions were:

- What are the common combinations of disease entities within multimorbidity among primary care patients?
- What is the level of severity in the leading combinations?
- What are the implications of our findings for future health care policy and practice?

## Methods

### Study design and participants

We undertook a cross sectional study in 40 primary care facilities (20 public and 20 private) in Odisha state of India. A two-stage clustered stratified random sampling method was adopted for recruiting health facilities. In the first stage, all 30 districts of the state were divided into two clusters i.e. economically well-developed (20) and less-developed (10), as per the state’s defined criteria[18]. From each cluster, districts were selected using stratified sampling methods, comprising four districts from less-developed and six from the developed districts. From each district, two community health centers (CHC) were randomly selected. For every CHC, a corresponding private facility in the vicinity was randomly included thus totaling to 20 private primary care facilities. We decided to include equal number of public and private facilities, as according to the national sample survey report, nearly 50% population in Odisha visited private facilities for any reported illness in addition or parallel to public health services. [19].

As we did not have studies from India on prevalence of multimorbidity, the required sample size was calculated based on our pilot study while validating the multimorbidity assessment tool. Considering that 23% of patients attending primary health care settings have multimorbidity[20], a sample of at least 1456 was needed to estimate this level of prevalence within relative precision error of 12.5% and design effect of 1.7. After accounting for 13% non- response based on our pilot experience[20], the final required sample size was 1670. It was decided to divide this sample number equally between private and public health care facilities as per the national sample survey report.

### Data collection

In view of unavailability of proper medical records, information was obtained through structured exit interviews with patients attending outpatient unit of public and private primary health care facilities. With no gold standard available to measure multimorbidity in India, we developed and validated a structured tool—Multimorbidity Assessment Questionnaire for Primary Care (MAQ-PC)[20]. We followed a two-phase iterative process to design this comprehensive tool. The first phase involved the development of the questionnaire, selecting the domains and their measurements, translating the questionnaire to local language for cultural adaptability, and testing its comprehensibility. The second phase entailed reliability and validity testing. The detailed methodology for development and validation of our tool is already described in another article.[20]. The socio-demographic section of the questionnaire elicited information on age, gender, place of birth, residence, ethnicity, religion, educational level, marital status, poverty status as per state guideline, current housing and household composition[21]. Multimorbidity assessment section of the questionnaire explored the presence of any of the 18 listed self-reported chronic diseases with three additional options of “any other” as reported by the patients (S1 Fig). The list of chronic conditions was prepared based upon the findings from our pilot study, extensive literature review and expert consultations. To evaluate depression, along with physician-diagnosed self-report, we included the Patient Health Questionnaire (PHQ-9) that is already validated for the Indian population[22]. The procedure for depression was different as compared to the assessment of the other chronic diseases, because the prevalence of depression in primary care in India is very low. For each of the reported chronic illnesses, severity was recorded by asking how much the condition gets in the way of their daily activities (e.g.: 0-not at all, a little, or 5- a great deal) [23]. Besides that for 10% of the patients, prescriptions were checked for types of drug being consumed and counterchecked with the wrapper of the drug/capsule.

The number of patients for each facility was calculated based on respective outpatient attendance. Adult patients (aged more than 18 years) were recruited by systematic random sampling and interviewed at exit from physician consultation. To avoid duplication, a unique identification number was given to patients. Interviews were conducted by four well trained field investigators with nursing background well versed with local language and patient history taking. Each interview spanned from 20 to 30 minutes.

### Data analysis

Three more chronic diseases were extracted from the additional list (S1 Fig) and added to the previous list of 18 thus totaling to 21 chronic diseases. We followed the prescribed guidelines for analysis of PHQ-9 towards diagnosing depression and a score of 10 or more was taken as a cutoff value for depression[24].

Analysis was carried out using sampling weight which was calculated taking account of the complex nature of the sample, i.e. different sampling fraction in each CHC or private facility and taking account of clustering by facility by using the ‘svy’ command in STATA (Version 12.0, Stata crop, TX). Because of two-stage design nature, the probability weight is calculated as f1f2, which means that the inverse of the sampling fraction for the first stage (i.e. using the formula N/n, where N = the number of elements in the population and n = the number of elements in the sample) is multiplied by the inverse of the sampling fraction for the second stage. Percentages in this paper are weighted and cluster adjusted. Confidence interval of the prevalence was calculated and chi-square test was performed for univariate analysis. We defined ‘multimorbidity’ as the presence of any two or more co-occurring chronic or long term diseases or conditions. Patterns (dyads and triads) were determined by employing a simple matrix approach exhaustive analysis of all possible combination of two or three co-morbid conditions using a simple descriptive statistical method[25].

We included those conditions which had a prevalence of more than 0.5% for analysis, below which the numbers of cases were negligible. Prevalence for single chronic condition (mono-morbidity) and leading combinations of two (dyad) and three chronic conditions (triad) was calculated for the whole study sample. Next, age specific prevalence of dyads and triads in men and women was analyzed separately after stratifying them into three age groups (18-39years, 40–64 years and ≥65 years) separately. Age adjusted severity score for dyads and triads was calculated by simple addition method and mean severity score was estimated for single diseases, dyads and triads with higher score indicating increasing functional limitation [23].

### Ethical consideration

The study adhered to the Declaration of Helsinki principles and was approved by the Institutional Ethics Committee of Public Health Foundation of India, New Delhi (Vide no. TRC-IEC-173/13). Respective physicians in charge of the health facilities were contacted and their permission was obtained in prior. Written informed consent was obtained from all respondents following an explanation of the study aims and procedures. Necessary steps were taken to preserve patient anonymity and confidentiality.

## Results

**Fig 1:**
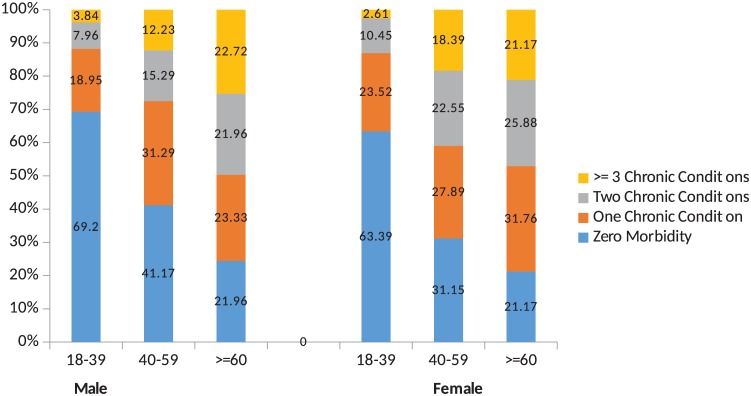
Number of chronic conditions in three age groups.

A total of 1670 primary care patients were approached, of which 1649 agreed to participate (response rate– 98%). The socio-demographic characteristics of the study participants are depicted in Table 1. Out of 1649, 921 and 728 were men and women respectively representing 6128 men and 4682 women after considering sampling weight. Nearly 60% of the participants were aged 40 or more and 28% were aboriginal. The average age of the study patients was 44.04 years [95% CI: 43.21–44.86]. The overall prevalence of multimorbidity was 28.3%. The mean number of chronic conditions and the prevalence of multimorbidity was more among women [32.5%] compared to men [25.1%] [X^2^ 14.23, p = 0.047] whereas, the mean severity score was higher among men [3.22, 95% CI 3.05–3.39] compared to women [3.15, 95%CI 2.97–3.33]. The total count of chronic conditions across age and gender is presented in Fig 1 which indicates there is increasing number of morbidities with age in both men and women.

**Table 1:** Prevalence of multimorbidity, mean morbidity score and mean severity score across socio-demographic variables.

Characteristics		Total (n = 1649) (Weighted %)	Percentage with multimorbidity (≥2conditions) Weighted % [95%CI]	Mean number of morbidity [Range: 0–21]	Mean severity score [Range 0–5]
Age Group*	18–29	373[22.6]	5.8[1.99–9.6]	0.26[0.06]	2.50[0.32]
	30–39	297[18.1]	22.2[15.1–29.4]	0.86[0.14]	2.91[0.28]
	40–49	346[20.5]	24.3[17.7–30.9]	1.06[0.06]	3.35[0.27]
	50–59	266[16.7]	36.2[27.9–44.5]	1.31[0.16]	3.19[0.27]
	60–69	236[14.6]	36.9[28.1–45.8]	1.47[0.18]	3.40[0.26]
	≥70	131[07.5]	44.4[33.0–55.8]	1.59[0.24]	3.47[0.40]
Gender*	Man	921[55.8]	25.1[22.1–28.0]	0.91[0.08]	3.19[0.12]
	Woman	728[44.2]	32.5[29.0–35.9]	1.08[0.08]	3.22[0.17]
**Place of living**	Rural	1493[90.4]	25.5[23.2–27.8]	1.00[0.06]	3.42[0.11]
	Urban	156[9.6]	28.5[27.8–29.3]	0.85[0.18]	3.21[0.09]
**Ethnicity**	Aboriginal	471[28.0]	27.7[26.3–29.2]	0.96[0.14]	3.45[0.11]
	Non aboriginal	1178[71.4]	28.5[27.6–29.4]	0.76[0.10]	3.51[0.10]
**Socio-economic status**	Below poverty line	1035[61.6]	28.8[27.8–29.7]	0.92[0.06]	3.30[0.17]
	Above poverty line	601[38.4]	27.5[26.2–28.8]	1.10[0.10]	2.99[0.15]
**Schooling**	No School	642[38.1]	35.0[33.7–36.3]	0.98[0.10]	2.98[0.09]
	Primary completed	514[30.7]	28.3[27.1–29.5]	1.17[0.12]	3.42[0.19]
	Secondary and above	493[31.1]	20.1[19.6–21.1]	0.94[0.12]	3.21[0.17]
**Marital Status**	Currently married	1321[79.8]	29.3[28.5–30.1]	0.25[0.08]	3.13[0.16]
	Currently not married	328[20.2]	24.3[22.0–26.6]	1.25[0.06]	3.65[0.18]
**Facility**	Public	849[61.0]	28.1[27.1–29.1]	1.02[0.08]	3.15[0.18]
	Private	800[39.0]	28.6[27.5–29.7]	0.93[0.08]	3.12[0.11]
**Total**		1649	28.3[25.9–30.7]	0.95[0.03]	3.15[0.05]

*The prevalence of multimorbidity across age group was adjusted for gender and across gender was adjusted for age, and for others, prevalence was adjusted for age and gender.

### Combination of co-morbid conditions

#### A. Distribution of single chronic condition (either alone or in combination)

**Fig 2:**
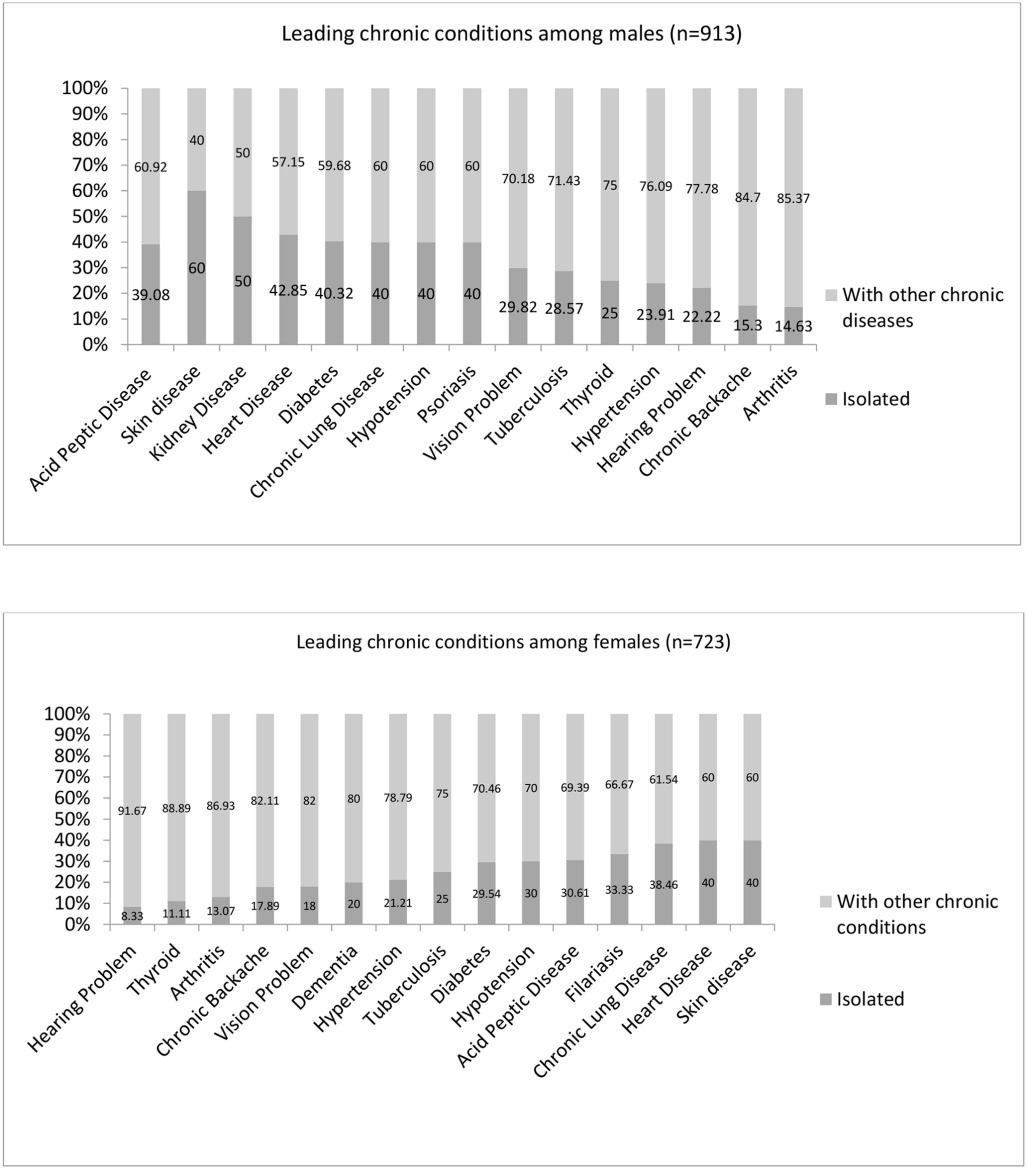
Prevalence of chronic condition (any and isolated) across gender.

The prevalence of single chronic condition either as isolated or comorbidity across gender is shown in Fig 2. In total, 439 individuals had isolated single chronic condition (men = 246, women = 193). Among men (n = 913) the leading chronic conditions were acid peptic disease (28.42%), hypertension (15.02%), arthritis (13.37%), chronic back ache (10.58%) and diabetes (6.71%) whereas for women (n = 723) acid peptic disease (33.71%), hypertension (18.16%), arthritis (17.92%), chronic back ache (13.10%) and visual impairment (6.85%) were found to be leading.

#### B. Two chronic conditions

Around 15% of study participants had two chronic conditions constituting 57.9% of all patients with multimorbidity. Table 2 denotes the age-wise distribution of the dyads for men and the same for women is given in Table 3. The dyads were almost similar for men and women. The combinations of acid peptic disease with arthritis/ chronic back ache/chronic lung disease were more common in younger age group i.e. 18–29 years older age groups had common combinations of hypertension + arthritis/ chronic lung disease/vision difficulty, and arthritis + chronic back ache.

**Table 2:** Prevalence (%) of dyads among men.

	18-39Yrs (N = 364) (Weighted %)	40-64Yrs (N = 425) (Weighted %)	≥65Yrs (N = 132) (Weighted %)	Total (N = 921)
APD+HTN	2.82	9.64	10.21	7.02
APD+ART	4.3	9	14.78	7.95
APD+CBA	3.71	7.92	10.6	6.63
ART+CBA	1.93	5.16	17.24	5.57
HTN+ART	0.91	4.12	11.82	3.92
HTN+DI	0.96	4	6.63	3.21
APD+VI	1.43	3.72	6.38	3.18
HTN+CBA	0.92	4.04	7.38	3.27
ART+VI	1.12	2.24	5.13	2.2
APD+DI	0.31	2.96	0.97	1.64
HTN+VI	0.18	1.36	4.69	1.36
ART+DI	0.5	0.99	3.16	1.1
APD+CLD	0.11	1.05	1.46	0.73
APD+DF	0	0.7	1.51	0.56
HTN+CLD	0.18	0.52	1.61	0.54

[APD- Acid peptic disease; HTN-Hypertension; ART-Arthritis; DI-Diabetes; CBA-Chronic backache; VI- Visual impairment; DF- Deafness; CLD- Chronic lungs disease]

**Table 3:** Prevalence (%) of dyads among women.

	18-39Yrs (N = 306) Weighted %	40-64Yrs (N = 337) (Weighted %)	≥65Yrs (N = 85) (Weighted %)	Total (N = 721)
APD+HTN	3.29	17.13	11.32	10.58
APD+ART	3.38	13.8	20.79	10.24
APD+CBA	4.83	10.48	10.14	8.05
ART+CBA	1.06	9.75	11.05	6.23
HTN+ART	0.48	7.36	12.73	5.1
APD+VI	1.18	6.8	5.07	4.22
ART+VI	0.54	4.48	10.02	3.48
HTN+CBA	0.31	3.96	5.34	2.58
HTN+DI	0	3.11	4.94	2.11
APD+DI	0.27	3.49	3.11	2.09
HTN+VI	0	3.34	3.72	1.97
ART+DI	0.46	1.76	6.2	1.74
APD+LD	0.62	2.73	0.77	1.6
APD+DF	0.59	1.24	2.68	1.14
HTN+CLD	0	1.84	0.77	0.93
APD+HYP	0.48	0.64	1.25	0.78
ART+CLD	0	0.29	2.85	0.47

[APD- Acid peptic disease; HTN-Hypertension; HYP-Hypotension; ART-Arthritis; DI-Diabetes; CBA-Chronic backache; VI- Visual impairment; DF- Deafness; CLD- Chronic lungs disease]

#### C. Triads (three chronic conditions)

**Fig 3:**
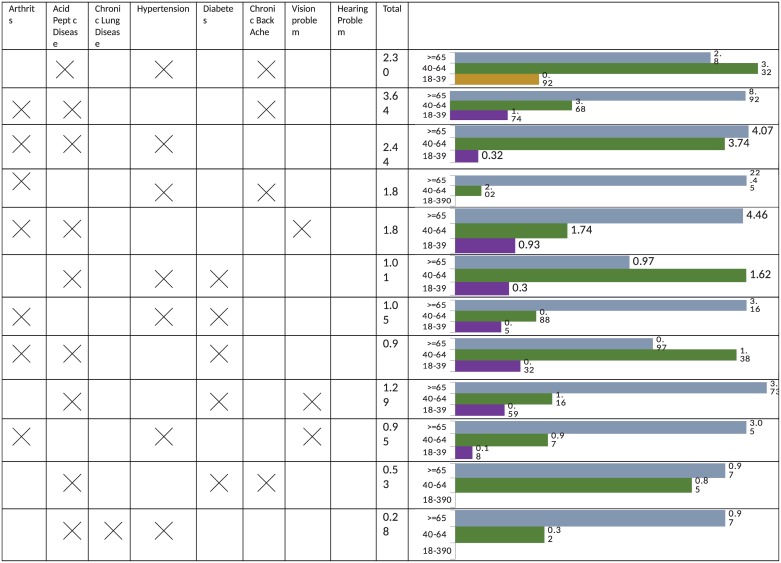
Prevalence (%) of triads among men.

**Fig 4:**
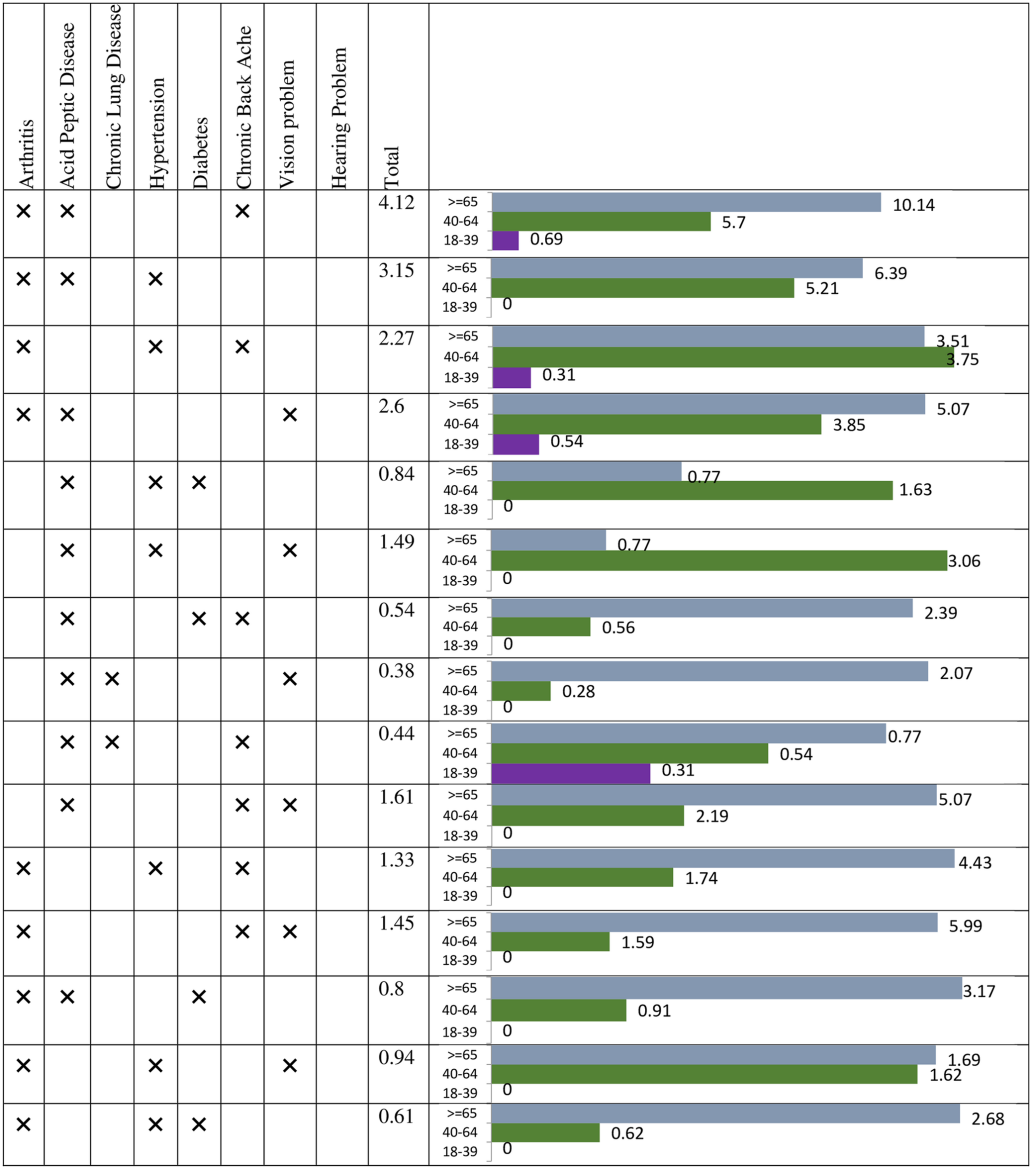
Prevalence (%) of triads among women.

Out of 437 multimorbid patients, 107 (24.48%) had three chronic conditions (men = 48, women = 59). Overall, the leading triads between men and women were similar. Participants in older age group 40–64 years showed the highest number of triads (Fig 3). The prevalence of acid peptic disease + arthritis + chronic backache was common in both younger and older age group in men. Among women, the leading triad was acid peptic disease + arthritis + chronic backache (31.24%) in the younger age group while in the older age group it was acid peptic disease + hypertension + arthritis (37.74%) (Fig 4)

#### D. Severity (functional limitation)

Mean severity score within mono-morbidities was highest for tuberculosis (TB) (2.81: 95% CI 2.40–3.22] followed by chronic lung disease [2.78: 95%CI 2.51–3.04] and heart disease [2.75: 95%CI 2.11–3.39]. The severity score was highest for heart disease [3.07: 95%CI 2.22–3.91], TB [2.94: 95%CI 2.50–3.37] and deafness [2.71: 95%CI 2.03–3.38] in men. Among women, arthritis [2.89: 95% CI 2.66–3.03], chronic lung disease [2.85: 95%CI 2.50–3.19] and chronic back ache [2.68: 95% CI 2.47–2.89] had leading mean severity score.

**Fig 5:**
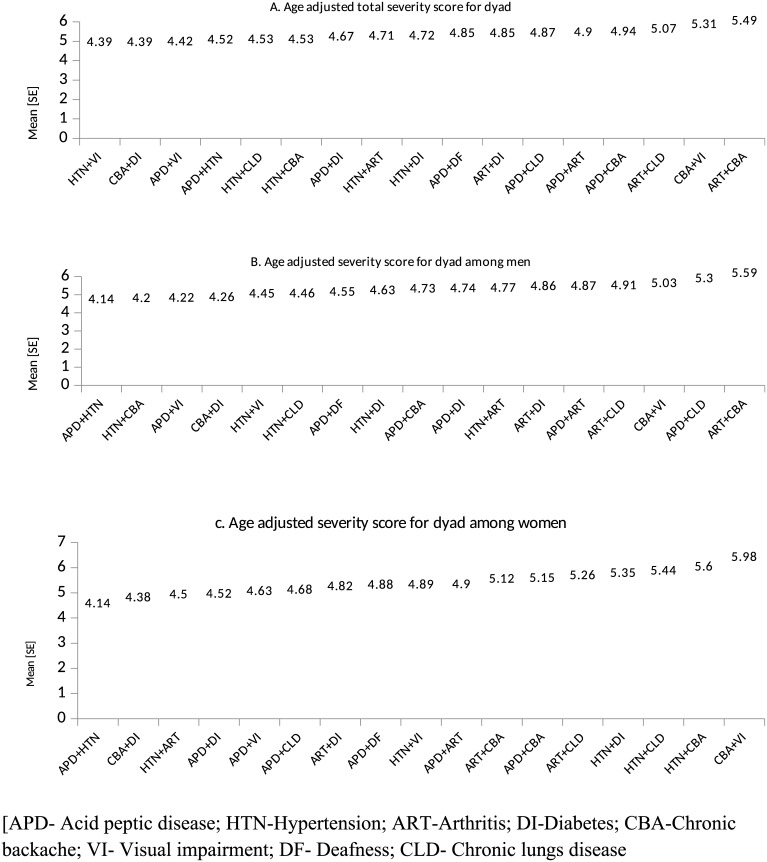
Severity score for dyad chronic conditions [Score range 0–10].

**Fig 6:**
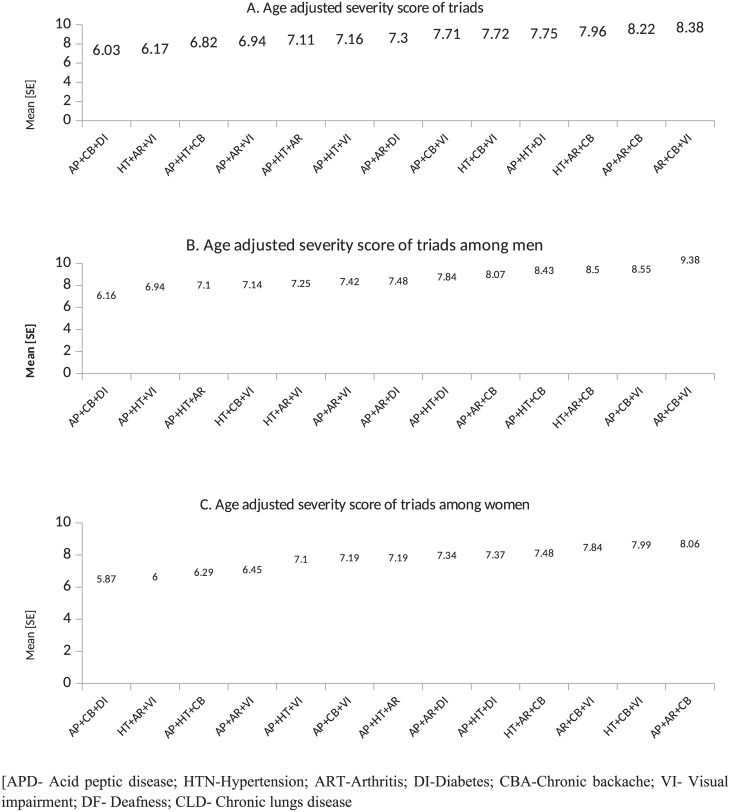
Severity score for triad chronic conditions.

Among the dyads, arthritis + chronic back ache [5.49: 95%CI 4.89–6.06], chronic backache+ vision impairment [5.31: 95%CI 3.81–5.81] arthritis + chronic lung disease [5.07: 95% CI [4.01–6.12], acid peptic disease + chronic backache [4.94: 95%CI 4.91–4.97] and acid peptic disease + arthritis [4.9: 95%CI 4.88–4.92] had highest burden score. Among men, arthritis + chronic backache [5.59: 95%CI 5.10–5.76] acid peptic disease + chronic lung disease [5.3: 95% CI 5.1–5.4] and chronic backache and vision impairment [5.03: 95%CI 4.98–5.08] were observed to have highest activity limitation (Fig 5). In women, chronic backache+ vision impairment, hypertension + chronic backache, hypertension + chronic lung disease and hypertension and diabetes had highest severity score (Fig 5). Within triads the leading conditions in terms of severity were found to be arthritis+ chronic backache+ vision impairment [8.38], acid peptic disease+ arthritis +chronic backache [8.22], hypertension + arthritis + chronic backache [7.96] and acid peptic disease + hypertension + diabetes [7.75] (Fig 6).

## Discussion

Multimorbidity, with its range of adverse consequences is arguably the greatest health care challenge globally. Such patients exhibit greater complexity, with different coexisting illnesses often simultaneously exacerbating the other thus leading to frequent physician consultation, longer hospitalizations, and higher health care costs with inferior quality of life[26]. Despite the global pervasiveness of multimorbidity limited studies have explored the combinations or pattern of diseases within the ambit of multimorbidity in LMIC[27]. Against this view, we examined both prevalence and patterns of multimorbidity among patients attending primary care in Odisha. We found multimorbidity to be common among primary care patients (proportion 28.9%) similar to western and other middle income countries[28–31]. For instance, a study in Ghana has identified 38.8% outpatients to have multimorbidity, while another study from Brazil has reported the prevalence to be 29.1% among adult population[30,31]. A recent study in South India has also demonstrated similar prevalence figures thus demonstrating the rising burden of multimorbidity in LMIC and no longer a restriction to wealthier countries[32].

### Frequently occurring chronic conditions

In our study, acid peptic disease (APD) was found to be the most common condition either alone or with other chronic diseases. This contra posits with most western findings where reports tend to be much lower (3%-8%). Hypertension and arthritis were the most frequently observed chronic condition. Available studies from LMIC have found hypertension and arthritis to be the most frequently occurring comorbidities [12,28,29]. However, one community survey done in India had identified the prevalence of APD to be considerably high which resonates with our findings[33,34]. Since both diagnosis and management of APD is done at primary care level most patients might be visiting primary care practice for such ailments. Arthritis, diabetes, hypertension and chronic lung diseases were found to be other leading chronic diseases, in congruence with other studies[15]. The increased proportion of hypertension and diabetes could be due to increased health literacy among patients, better symptom appraisal by primary care physicians and availability of diagnostic facilities under the national non communicable disease control program which primarily focuses on diabetes and hypertension. We found high prevalence of arthritis and lung diseases in primary care in coherence with other studies from India as well as LMIC[29–31,35]. However, the national NCDs control program is yet to incorporate these conditions in their priorities.

### Combination of chronic conditions

We classified the combinations (dyads and triads) and assessed their clinical relevance by the plausibility of disease interactions. The coexisting conditions may be concordant that emanate from the same pathophysiology, or discordant conditions arising from separate pathophysiologies[36]. Multimorbidity patterns have traditionally been classified into three cumulative categories: simple co-/multimorbidity (the co-occurrence of diseases, whether coincidental or not), associative co-/multimorbidity (statistical association, not or not known to be causal); and causal co-/multimorbidity (implying a causal relation among co-occurring diseases) [2].

Despite the inclusion of 21 diseases in multimorbidity assessment, most dyads (having proportion of more than one percent) were confined to 10 conditions. From 16 dyads identified, two were causal, eight were simple multimorbidity and six were associative in nature. Acid peptic diseases was present in 50% dyads while hypertension and arthritis each contributed to 40% followed by 20% dyads having diabetes, chronic backache and chronic lungs diseases as a comorbidity.

Given the absence of similar studies from primary care settings it is challenging to compare our findings within South Asia. However, our proportion of causal comorbidities was near similar to that observed in western countries[37,38]. The coexistence of hypertension and diabetes has been widely documented. Both share similar risk factors and clustered under metabolic syndrome[39]. Contrastingly, there is scanty evidence available on concurrent presence of hypertension and chronic lung diseases[40]. One plausibility could be the bronchospasm induced by prolonged use antihypertensive medications[41]. To explore such association future studies need to be done. Mostly, the therapeutic guidelines are available for individual diseases especially, hypertension, diabetes and asthma. Given the commonly occurring dyads, it is imperative that the primary care protocols should be designed to accommodate causal co- morbidities as well.

Associated comorbidities arise because of the therapeutic impacts or consequences of the primary or index diseases. In our study, acid peptic disease was predominantly coexistent with arthritis, chronic back ache and hypertension. The relationship between musculoskeletal diseases and APD is well evident[42]. Non-Steroidal Anti Inflammatory Drugs (NSAIDs) known to have both upper and lower gastrointestinal tract erosions are routinely prescribed for a variety of musculoskeletal complaints such as arthritis, chronic back ache, and short-term management of pain[43,44]. More so over the counter prescription of NSAID is a common practice in India which could have been another contributory reason[45,46]. Similarly, calcium channel blockers and beta blockers prescribed for hypertension may aggravate or precipitate gastrointestinal reflux leading to increased prevalence of APD[47].

Acid peptic disease was coexisting with deafness, chronic lung diseases and vision impairment thus sharing 50% of the total discordant pattern. Another major discordant combination was arthritis or chronic back ache with hypertension. Such combinations could be resulting from the interplay of multiple factors such as increase in age, obesity and/or sedentary life style. The knowledge of chronological occurrence of the diseases could have been partially able to explain the pattern. For example, limitation of physical activities in arthritis might predispose hypertension; on the other hand, increased body weight or sedentary life style can potentiate the risk of arthritis and hypertension[48]. Similarly, presence of vision impairment in hypertension either be induced by hypertension or related to age. Since we had not collected information on exact reasons of vision problems at primary care facilities, it is difficult to ascertain the probable causes. Further studies should strive to identify any plausible biological or etiological factors underlying such discordant patterns.

### Severity of the pattern

We estimated age adjusted severity score for all dyads and triads. The mean severity score among dyads ranged from 4.39 to 5.49 [normal range 2–10] and 6.03 to 8.38 for triads [normal range 3–15]. Even though the disease count was two, the variation in age adjusted severity score explains the importance of typology of co morbidities. For example we found that, in dyads, presence of arthritis, chronic back ache and chronic lung diseases with other conditions inflates the burden score indicating functional limitations. Diseases having clear manifestations of physical symptoms impact the activity of daily living of a patient more, than diseases like hypertension and diabetes. It is evident that, such physical symptoms like pain in joints or breathing problem affects an individual’s activities thus impairing the quality of life[49]. Our study also reveals the high functional limitation accruing from musculoskeletal disorders, which advocates for availability for the musculoskeletal care in primary care setting in India.

### Implication for future research, health care policy and practice

In persons with multimorbidity, there exists co-occurrence of diseases beyond chance, which clinicians need to take into account in their daily practice. We found few emerging, unexpected non-random associations that may not logically follow previous biomedical research but that offer promising directions for future research. Some pathological mechanisms behind the identified clusters are well-known (for instance diabetes and hypertension; diabetes and cardiovascular diseases); other combinations (COPD and arthritis) need further clarification to identify possible preventative strategies. Even though age is seen to play a crucial role in determining the combination of diseases, the variety of patterns in reported in the younger age group necessitates future research to identify the biomarkers or linking factors. Regarding the clinical applicability of these findings, apart from generating new hypotheses on possible shared pathophysiological pathways among specific diseases, research on multimorbidity patterns may orient prevention and optimal management (including treatment decisions) for individuals suffering from a given index disease who develop successive conditions. Moreover, certain combinations were found to be associated with higher functional decline, underscoring the importance of global assessment of multimorbid patients for functional limitations. Further research, preferably of longitudinal design, should investigate the possible causal underlying mechanisms, trajectory, clinical impact and the financial implications of these associations so as to predict health care needs and reduce the adverse health impacts in patients.

Effective management of multimorbidity is challenging in primary care practice[50]. Physicians and patients can be overwhelmed by the need to address comorbid chronic conditions in addition to patients’ disease specific treatment goals. Therefore while managing concurrent diseases it is imperative to consider severity (functional limitation) for effective control of the disease and improve patients’ quality of life. Current national NCD program should design customized or tailor made disease or case management protocols for patients with multiple morbidities, as most of the patients might be consulting individual providers thus resulting in fragmented care and suboptimal outcomes. Firstly, specific treatment guidelines or patient education programs could be developed for persons with high-frequency clusters or with clusters of diseases particularly difficult to be treated. Further, evidence based research on better care model for patients with multimorbidity to reduce the potential for drug–drug and drug–disease interactions should be initiated.

### Strengths and limitations

Our work for the first time reveals the multimorbidity patterns present among primary care patients and indicates the complex heterogeneous problems of the same in India. However, we restricted the patients to primary settings and the magnitude may be more or less in secondary and tertiary care facilities. Further, being of cross sectional nature, the causal and trajectory cannot be assessed. We ascertained multimorbidity through self-reports which may be prone to recall bias. Nonetheless, the validity and reliability of self-report is well established in our previous study[20]. Despite these limitations, our study provides a very timely contribution to the current understanding of clustering and severity of multiple chronic diseases and, in particular, provides important insights into approaches to designing care strategies for such patients in Indian context. Findings from our study have significant implications for program planners, general practitioners, and policy-makers, valuable information relating to comprehensive and coordinated management of chronic disease and the delivery of primary care services in India.

## Supporting information

S1 Fig List of chronic diseases included in morbidity assessment protocol. (DOCX)
