# Supplementary material for: Pattern and severity of multimorbidity among patients attending primary care settings in Odisha, India
Source: PLoS One. 2017 Sep 14;12(9):e0183966. doi: 10.1371/journal.pone.0183966 (PMC5598947; doi:10.1371/journal.pone.0183966)
Supplement: S1 Fig — (DOCX) [file pone.0183966.s001.docx]

List of chronic diseases included in morbidity assessment protocol

| Diseases included | | | |
| --- | --- | --- | --- |
| Sl no | Name | Questions asked for Self reported doctor diagnosis | Questions asked for Symptomatic validation |
|  | Diabetes | Yes | No |
|  | Hypertension | Yes | No |
|  | Arthritis | Yes | Yes* |
|  | Acid peptic disease | Yes | No |
|  | Asthma | Yes | No |
|  | Heart disease | Yes | Yes* |
|  | Stroke | Yes | Yes* |
|  | Chronic kidney disease | Yes | Yes*(Dialysis) |
|  | Chronic liver disease(Alcohol) | Yes | Yes* |
|  | Chronic back ache | Yes | Yes* |
|  | Tuberculosis | Yes | Yes(Currently under antiTB drugs) |
|  | Filariasis | Yes | No |
|  | Visual difficulty | Yes | Yes* |
|  | Deafness | Yes | Yes* |
|  | Cancer | Yes | No |
|  | Dementia | Yes | Yes* |
|  | Epilepsy | Yes | Yes* |
|  | Thyroid | Yes | No |
| Others | |  |  |
|  | Hypotension |  |  |
|  | Anemia |  |  |
|  | Psoriasis |  |  |
| Depression was screened by using (PHQ-9). | | | |
| *Validated on pilot study  In the current article we have considered self reported doctor diagnosis for estimation of prevalence of individual chronic diseases and multimorbidity | | | |

Dyad Causal: (hypertension + diabetes) and (hypertension + chronic lung disease),

Dyad Simple - (acid peptic disease + hypertension, arthritis + chronic backache, hypertension+ arthritis, arthritis+ diabetes, acid peptic disease + deafness, hypertension+ chronic back ache, arthritis + vision impairment, hypertension + vision impairment and acid peptic disease+ chronic lung diseases) and

Dyad Associative - (acid peptic diseases + arthritis, acid peptic disease + chronic back ache, acid peptic disease + diabetes and acid peptic disease + hypertension ).

Appendix-II (Selection of study sites)

KBK+ Districts (10)

90

Non KBK Districts (20)

4Districts

6Districts

2 CHCs and 2 private hospitals from each district

2 CHCs and 2 private hospitals from each districts

In total 20 CHCs and 20 private hospitals were included in the study

Odisha State
